# Association between serum vitamin D levels and sensitivity to thyroid hormone indices: a cross-sectional observational study in NHANES 2007–2012

**DOI:** 10.3389/fendo.2023.1243999

**Published:** 2023-09-05

**Authors:** Si Chen, Wei Yang, Zhen Guo, Xiaofei Lv, Yandun Zou

**Affiliations:** Department of Internal Medicine, Guangdong Women and Children Hospital, Guangzhou, Guangdong, China

**Keywords:** vitamin D, sensitivity to thyroid hormone indices, thyroid feedback quantile-based index, free triiodothyronine/free thyroxine, NHANES

## Abstract

**Objective:**

We designed this study to determine whether there is a link between vitamin D levels and sensitivity to thyroid hormone and to provide a new perspective for studying the relationship between vitamin D and thyroid disease.

**Methods:**

Our study included 8,126 participators from the National Health and Nutrition Examination Survey (NHANES) database between 2007 and 2012. We used weighted multiple linear regression models to enquire the connection between serum vitamin D levels and thyroid hormone sensitivity indicators, including the following: Thyroid-stimulating hormone index (TSHI), Free Triiodothyronine/Free thyroxine (FT3/FT4), Thyroid Feedback Quantile-based Index (TFQI), and Thyrotroph Thyroxine Resistance Index (TT4RI). Finally, we used constrained cubic splines to explore possible nonlinear relationships. All data cleaning and statistical analyses were performed using R software.

**Results:**

The final Results were reached after adjusting for various confounding factors. We found a U-shaped relationship between TFQI and serum vitamin D, and the lowest TFQI appeared when the serum vitamin D concentration was 25.77ng/ml. However, an inverse U-shaped relationship was found between FT3/FT4 and vitamin D levels. When the serum vitamin D concentration was 25.43ng/ml, the ratio of FT3/FT4 was the highest.

**Conclusion:**

In the US population, our study concluded that FTQI and FT3/FT4 were U-shaped or inverse-U-shaped with serum vitamin D levels respectively after several adjustments. Therefore, FTQI and FT3/FT4 are considered indicators of the complex relationship between thyroid hormone resistance and vitamin D metabolism. In the future, more complex prospective investigations are needed to confirm these findings and find a causal link between them.

## 1 Introduction

Vitamin D is a basic nutrient for human health which contains two bioequivalent forms: Vitamin D3 and Vitamin D2. In humans, Vitamin D2 is obtained by ingesting food, especially dietary vegetables, and taking oral supplements. Vitamin D3 can be obtained in several ways: synthesized by skin exposure to UV-B radiation, food intake such as various Marine fish, and oral supplements (1). After obtained from the intestine by ingesting food or from the skin by exposing to ultraviolet-B radiation, vitamin D is immediately transported to the liver, where it is converted to 25-hydroxyvitamin D, and then continues to circulate in this form to the kidney. Eventually, 25-hydroxyvitamin D is catalyzed to 1,25-dihydroxyvitamin D and exerts its physiological effects in this form (2). Vitamin D has been valued for its following effects: maintaining the balance of serum calcium and phosphorus, promoting the absorption of intestinal calcium, and affecting bone growth through the activity of osteoblasts and osteoclasts. Numerous studies have been conducted in the past to explore the relationship between vitamin D deficiency and Skeletal diseases such as osteomalacia, fractures, and osteoporosis, and their close relationship has been well recognized now. Yet evidence is mounting that low levels of vitamin D are related to autoimmune diseases, cardiovascular disease, metabolic syndromes, cancers, and infection (3). However, vitamin D deficiency remains a common health problem that deserves our further research.

In clinical practice, we evaluate the thyroid function status of patients by measuring serum TSH, FT3 and FT4 levels. However, we cannot fully understand thyroid hormone homeostasis with these three indicators alone (4). Some scholars have proposed the concept of thyroid hormone sensitivity index, which consisted of the following four specific indicators: TSHI, TT4RI, TFQI, and FT3/FT4, hoping to help researchers better understand the body’s thyroid hormone status. TSHI, TT4RI, and TFQI can quantitatively evaluate Pituitary-thyroid axis function. Through the FT3/FT4 ratio, we can probably know the efficiency of converting peripheral FT4 into FT3, so as to understand the peripheral sensitivity of Thyroid hormones (5–9). Increasing number of surveys have been conducted on the correlation between thyroid hormone sensitivity and other diseases in recent years. Researchers have confirmed that Thyroid hormones sensitivity indicators are related to metabolic syndrome, including cardiovascular disease, dyslipidemia, insulin resistance, and type 2 diabetes (9, 10).

In addition to bone health, vitamin D has many other functions, of which the two most important non-skeletal functions include: immunomodulatory and anti-inflammatory functions. Therefore, it may help prevent or improve inflammation and participate in the regulatory pathways of immune mediated tissue injury. The correlation between autoimmune thyroid disease and vitamin D was controversial. A systematic review exploring the relationship between autoimmune thyroid disease and vitamin D levels demonstrated that Grave’s disease and Hashimoto thyroiditis were both relevant to lower levels of vitamin D (11). However, some scholars had to admit that their researches failed to confirm the correlation between autoimmune Thyroiditis and vitamin D (12). No research has been found on the relationship between sensitivity to thyroid hormone indices and vitamin D. For that reason, we aimed to investigate the relationship between serum vitamin D levels and thyroid hormone sensitivity in US population using data from a public database (NHANES) in the present study.

## 2 Materials and methods

### 2.1 Population included in the study

**Figure 1:**
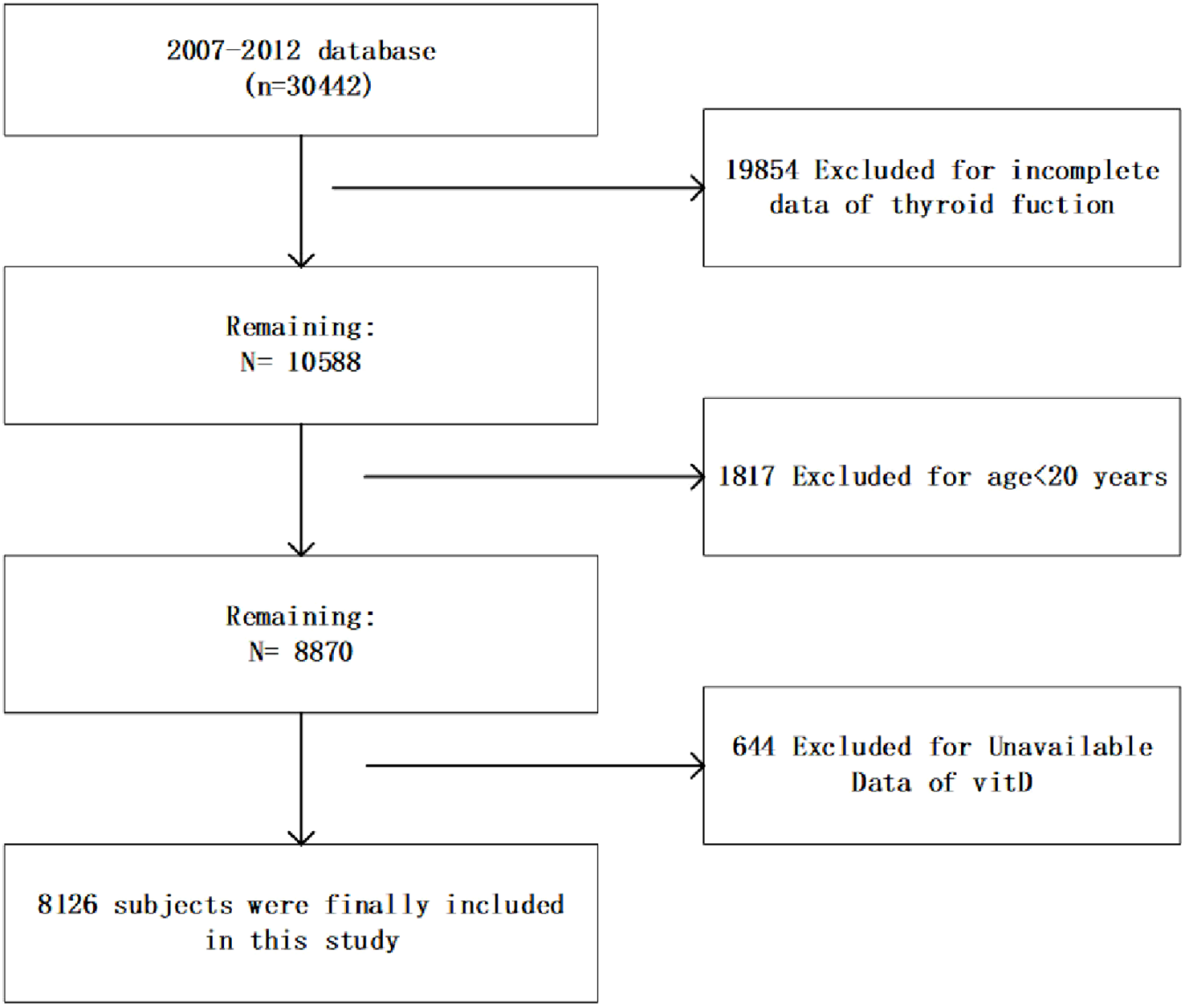
Flowchart of the study population.

This cross-sectional study used database from the NHANES, an ongoing study conducted by the Centers for Disease Control (CDC) since the 1980s, with the aim of assessing the nutritional and health status of American residents and developing health guidelines. We collected demographic, questionnaire, examination and laboratory information from three consecutive NHANES cycles from 2007 to 2012. As shown in **Figure 1**, this study included 8126 subjects who had complete information on serum vitamin D levels and thyroid function aged >20 years. Informed consents prepared by the NCHS Ethical Review Board were obtained from all participants.

### 2.2 Assessment of Vit D

Concentrations of Serum 25(OH)D were detected at the Division of Laboratory Sciences, National Center for Environmental Health, Centers for Disease Control and Prevention, Atlanta, GA, using a method called ultra-high performance liquid chromatography-tandem mass spectrometry. In our study, we first determined the classification nodes based on the guidelines of the Endocrine Society, and then divided the subjects into three categories according to the serum 25 (OH) D levels. Serum 25(OH)D concentrations <20 ng/mL was regarded as VD deficiency, serum 25(OH)D concentrations between 20 and 29.9 ng/ml was regarded as VD insuficiency and serum 25(OH)D concentrations≥ 30.0ng/mL was regarded as VD suficiency (11).

### 2.3 Assessment of thyroid function

The TT3, FT3 and TT4 were detected using a competitive binding immunoenzymatic assay. The assay of Free T4 was a two-step enzyme immunoassay. The Free T4 assay was a two-step enzyme immunoassay. The detection of sensitive human thyroid stimulating hormone was performed by a two-site “ sandwich “ immunoenzyme test. TgAb and TPOAb (IU/mL) were assessed by a sequential two-step immunoenzymatic “sandwich” assay, while Tg levels (ng/ml) were detected by a simultaneous one-step “sandwich” assay.

### 2.4 Sensitivity to thyroid hormone indices assessment

FT3/FT4, TT4RI, TFQI and TSHI constitute thyroid hormone sensitivity indicators, and their calculation formulas are as follows:

(1) FT3/FT4 =FT3/FT4 (13)
(2) TT4RI =FT4 (pmol/L) ∗ TSH (mIU/L) (13)
(3) TFQI = cdfFT4 − (1−cdfTSH) (9)
(4) TSHI = ln TSH (mIU/L) + 0.1345∗ FT4 (pmol/L) (6)

Sensitivity of peripheral tissue to thyroid hormone raised with the augment of FT3/FT4 ratio. The central thyroid hormone sensitivity decreased with the augment of TSHI and TT4RI index. TFQI is an indicator used to assess the central thyroid hormone sensitivity, and it was computed based on the empirical cumulative distribution function, with values ranging from -1 to 1. Referring to conclusions drawn from the former studies, we defined TFQI=0 as the reference standard, indicating that the HPT axis had a normal sensitivity to FT4 changes. A value less than zero indicated that the HPT axis had a higher sensitivity to FT4 variations, while a value greater than zero represented that human HPT axis responded slowly to FT4 variations (9).

### 2.5 Covariates

In accordance with previous similar studies, we believed that the below variables may affect our statistical results, so we identified them as confounders in this study: gender, age, race, educational background, marriages, Family income to poverty ratio, drinking behavior, smoking behavior, physical activities, BMI, diabetes, hypertension, glycohemoglobin, albumin (g/dL), serum calcium(mg/dL), blood urea nitrogen (mg/dL), cholesterol(mg/dL), triglycerides(mg/dL), phosphorus (mg/dL), serum uric acid (mg/dL)and creatinine (mg/dL) (12). The race in our study included Mexican Americans, non-Hispanic whites, other Hispanics, non-Hispanic blacks, and other races. Educational background included the following four categories: college graduate or above, some college, high school grade, 9-11th grade, and less than 9th grade. Marriages were divided into married, widowed, divorced, separated, never married, living with partner. drinking behavior and smoking behavior were both classified as never, former and current (14). Physical activity was defined as yes (vigorous recreational activities or moderate recreational activities) and no (no moderate recreational activities) (14). The classification of BMI was defined as follows: underweight (<18.5), Normal weight (≥18.5, <25), overweight (≥25, <30) and obese (≥30) (13). Patients with the following conditions were defined as “hypertension”: systolic blood pressure ≥ 140 or/and diastolic blood pressure ≥ 90mmHg or having a history of hypertension (15). Participants with one of the following conditions were considered to have diabetes: a history of diabetes, use of insulin or oral hypoglycemic agents (16).

### 2.6 Statistical analyses

Following NHANES’s suggestion, sample weights based on stratified, multi-stage probabilistic sampling design were used for all analyses. Continuous variables were displayed as mean standard deviation, and categorical variables were shown as percentage of subjects. According to different levels of serum vitamin D, the study subjects were divided into three groups. P values were calculated using the weighted chi-square test and the linear regression model. We used a weighted multiple linear regression model to investigate the relationship between serum vitamin D levels and sensitivity to thyroid hormone indices in three different models. Model 1 adjusted no variable. Model2 adjusted for the following variables: age, race, gender, family income to poverty ratio, educational background, marriages. Model 3 further adjusted for age, race, gender, family income to poverty ratio, educational background, marriages, physical activities, BMI, smoking behavior, drinking behavior, diabetes, hypertension, glycohemoglobin, serum urea nitrogen, cholesterol, triglycerides, serum calcium, phosphorus, creatinine, serum uric acid, and albumin.

Restricted cubic spline with three knots at the 10th, 50th, 90th were conducted to explore the nonlinear relationship between serum vitamin D levels and sensitivity to thyroid hormone indices after adjusting for cofounding factors, including age, race, gender, educational background, marriages, family income to poverty ratio, physical activities, BMI, smoking behavior, drinking behavior, diabetes, hypertension, glycohemoglobin, serum urea nitrogen, albumin, phosphorus, serum calcium, cholesterol, triglycerides, serum uric acid, and creatinine.

All analyses in the study were weighted to represent the US population. When the P-value less than 0.05, we considered the difference to be statistically significant. We Performed all statistical analyses using R (version 4.2.2).

## 3 Results

### 3.1 Baseline characteristics of the participants

The weighted demographic and socioeconomic characteristics grouped into three categories based on serum vitamin D levels were presented in **Table 1**. The weighted clinical characteristics divided into three groups based on levels of vitamin D were presented in **Table 2**. The total number of subjects contained in this research is 8126.

**Table 1:** Weighted demographic characteristics of study population based on serum vitamin D levels.

Characteristics	Deficiency (<20ng/ml)	Insufficiency (>=20, <30 ng/ml)	Sufficiency (≥30 ng/ml)	P value
Gender (%)				<0.001
Male	46	53.9	44.3	
Female	54	46.1	55.7	
Age(years)	43.75 ± 16.29	46.13 ± 16.36	50.70 ± 16.93	<0.001
Race (%)				<0.001
Mexican American	14.2	9.6	2.5	
Other Hispanic	8.2	7.0	3.0	
Non-Hispanic White	39.1	69.8	87.0	
Non-Hispanic Black	26.9	7.1	3.2	
Other Race	11.6	6.5	4.3	
Education level (%)				<0.001
Less than 9th grade	8.4	7.1	4.3	
9–11th grade	15.1	11.6	10.5	
High school grad	24.4	21.7	22	
Some college	31	30.8	30.5	
College graduate or above	21.1	28.8	32.7	
Marital status (%)				<0.001
Married	45.6	57.8	59.4	
Widowed	5.5	5.8	6.8	
Divorced	11	8.2	11	
Separated	3.4	2.5	1.7	
Never married	24.8	17.6	14.2	
Living with partner	9.7	8.2	6.9	
Family income to poverty ratio	2.47 ± 1.62	2.98 ± 1.62	3.30 ± 1.62	<0.001
Drinking behavior (%)				<0.001
Never	26.3	19.6	16.7	
Former	9.0	9.2	9.7	
Current Missing	54.0 10.7	63.4 7.9	65.8 7.8	
Smoking behavior (%)				<0.001
Never	58.5	55.5	51.6	
Former	16.9	24.1	30.1	
Current	24.6	20.4	18.3	
Physical activity (%)				<0.001
Yes	41.6	53.6	61.9	
No	58.4	46.4	38.1	

Mean ± SD for continuous variables: the P value was calculated by the weighted linear regression model. (%) for categorical variables: the P value was calculated by the weighted chi-square test.

**Table 2:** Weighted clinical characteristics of study population based on serum vitamin D levels.

Characteristics	Deficiency (<20ng/ml)	Insufficiency (>=20, <30 ng/ml)	Sufficiency (≥30 ng/ml)	P value
BMI (%)				<0.001
Underweight(<18.5)	1.9	1.1	2.3	
Normal weight(≥18.5,<25)	24.2	25.5	37.4	
Overweight(≥25,<30)	27.5	36.0	34.7	
Obese(≥30) Missing	44.9 1.5	36.5 1.0	24.7 0.8	
Hypertension (%)	31.0	30.2	34.0	0.167
Diabetes (%)	12.5	9.8	8.0	0.004
Glycohemoglobin	5.78 ± 1.13	5.61 ± 0.89	5.54 ± 0.66	<0.001
Albumin (g/dL)	4.22 ± 0.34	4.30 ± 0.32	4.32 ± 0.34	<0.001
Blood urea nitrogen (mg/dL)	11.96 ± 5.34	12.94 ± 4.86	13.76 ± 5.29	<0.001
Total calcium (mg/dL)	9.37 ± 0.36	9.40 ± 0.35	9.45 ± 0.35	<0.001
Cholesterol (mg/dL)	192.85 ± 42.22	195.63 ± 41.78	197.38 ± 41.10	0.043
Triglycerides (mg/dL)	154.09 ± 131.29	165.30 ± 147.53	145.73 ± 113.17	0.011
Creatinine (mg/dL)	0.86 ± 0.38	0.87 ± 0.26	0.89 ± 0.31	0.008
Phosphorus (mg/dL)	3.73 ± 0.55	3.75 ± 0.57	3.78 ± 0.57	0.12
Uric acid (mg/dL)	5.47 ± 1.45	5.50 ± 1.4	5.34 ± 1.40	0.018
FT4 (pmol/L)	10.50 ± 2.22	10.32 ± 1.97	10.50 ± 2.19	0.031
FT3 (pmol/L)	4.97 ± 0.65	4.91 ± 0.86	4.78 ± 0.65	<0.001
FT3/FT4	0.49 ± 0.10	0.49 ± 0.12	0.47 ± 0.14	<0.001
TSH (mIU/L)	1.98 ± 3.79	2.21 ± 4.70	1.97 ± 1.73	0.124
TSHI	1.79 ± 0.73	1.83 ± 0.70	1.86 ± 0.72	0.049
TT4RI	19.36 ± 19.93	20.78 ± 25.46	20.34 ± 17.97	0.201
TFQI	-0.02 ± 0.26	-0.03 ± 0.25	-0.01 ± 0.27	0.07
TPOAb (IU/mL)	20.12 ± 92.44	19.94 ± 87.15	25.11 ± 98.49	0.331
TgAb (IU/mL)	10.67 ± 84.77	11.67 ± 102.70	7.81 ± 68.94	0.321
Tg (ng/mL)	16.89 ± 31.40	15.43 ± 40.83	15.10 ± 25.77	0.286

Mean ± SD for continuous variables: the P value was calculated by the weighted linear regression model. (%) for categorical variables: the P value was calculated by the weighted chi-square tes.t

Several demographic factors were found to differ significantly among the three groups. Participants in the insufficient and adequate groups had a higher probability to be seniors, people with higher education, non-Hispanic white, married, and have a higher ratio of family income to poverty than those in the deficient group. At the same time, referring to smoking behavior, physical activities, and drinking behavior, the three groups differed significantly.

Comparison of clinical characteristics among the three groups also differ significantly in most of the factors. Normal-weight participants were more likely to be in the Sufficiency group. Nearly 12.5% of the deficiency group had diabetes, compared to 8.0% and 9.8 in the Sufficiency and insufficiency groups. Meanwhile, there were significant differences in glycohemoglobin, albumin, serum calcium, blood urea nitrogen, cholesterol, creatinine, triglycerides, serum uric acid, and phosphorus among the three groups. In addition, we also found significant differences in TSHI, FT4, FT3, and FT3/FT4 levels among the three groups.

### 3.2 Whether serum vitamin D levels correlate with sensitivity to thyroid hormone indices?

We found that there was no linear correlation between FT3/FT4, TSHI, TFQI, TT4RI and serum vitamin D levels after adjusting confounding factors. The outcomes of weighted multiple linear regression analyses are shown in **Table 3**. Furthermore, after transforming serum vitamin D levels to a categorical variable (quartiles), We still don’t see a linear trend between FT3/FT4, TSHI, TFQI, TT4RI and serum vitamin D levels (**Table 4**).

**Table 3:** Associations between sensitivity to thyroid hormone indices and serum vitamin D levels (ng/ml).

β(95% CI)P	TSHI	FT3/FT4	TFQI	TT4RI
model1	0.0021(-0.0005 0.0047) 0.106	-0.0008(-0.0012 -0.0005) P<0.001	0.0007(-0.0003 0.0017) 0.145	0.0390(-0.0392 0.1172) 0.321
model2	0.0004(-0.0020 0.0028) 0.748	-0.0004(-0.0007 -0.0001) 0.0205	0.0003(-0.0007 0.0012) 0.579	-0.0126(-0.0859 0.0607) 0.729
model3	0.0001(-0.0023 0.0034) 0.682	-0.0002(-0.0006 0.0001) 0.1700	0.0003(-0.0008 0.0014) 0.642	-0.0051(-0.0834 0.0733) 0.677

Model 1: adjusted no variable.

Model 2: adjusted for the following variables: age, race, gender, family income to poverty ratio, educational background, marriages.

Model 3: adjusted for age, race, gender, family income to poverty ratio, educational background, marriages, physical activities, BMI, smoking behavior, drinking behavior, diabetes, hypertension, glycohemoglobin, serum urea nitrogen, cholesterol, triglycerides, serum calcium, phosphorus, creatinine, serum uric acid, and albumin.

**Table 4:** β(95%CI) for thyroid hormone sensitivity indices according to quartiles of vitamin D levels.

	TSHI	FT3/FT4	TFQI	TT4RI
VID	β(95% CI)	β(95% CI)	β(95% CI)	β(95% CI)
Q1	Ref.	Ref.	Ref.	Ref.
Q2	0.001(-0.074 0.103)	-0.003(-0.011 0.005)	0.002(-0.021 0.026)	0.791(-1.711 3.293)
Q3	0.036(-0.035 0.108)	0.002(-0.009 0.014)	-0.007(-0.037 0.022)	1.633(-0.105 3.370)
Q4	0.026(-0.058 0.111)	-0.002(-0.012 0.009)	0.002(-0.028 0.033)	-0.182(-2.137 1.773)
P-trend	0.51	0.96	0.94	0.62

Analyses were adjusted for age, race, gender, family income to poverty ratio, educational background, marriages, physical activities, BMI, smoking behavior, drinking behavior, diabetes, hypertension, glycohemoglobin, serum urea nitrogen, cholesterol, triglycerides, serum calcium, phosphorus, creatinine, serum uric acid, and albumin.

**Figure 2:**
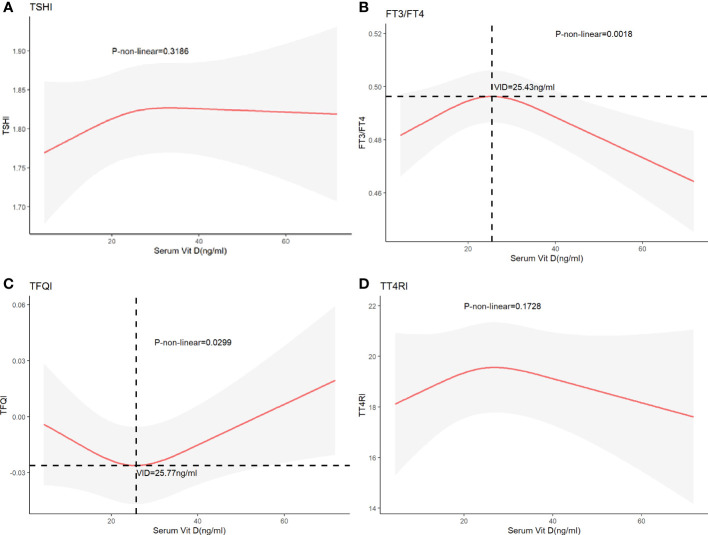
The nonlinear association **(A)** between TSHI and vitamin D, **(B)** FT3/FT4 and vitamin D, **(C)** TFQI and vitamin D, and **(D)** TT4RI and vitamin D.

The restricted cubic splines were further adopted to explore the nonlinear association between FT3/FT4, TSHI, TFQI, TT4RI and vitamin D levels. Outcomes are shown in **Figures 2A–D**. After adjustment for possible confounders, a U-shaped relationship was noticed between TFQI and serum vitamin D levels, who had the lowest TFQI when the serum vitamin D concentration was 25.77ng/ml. An inverse U-shaped relationship was found between FT3/FT4 and serum vitamin D levels. When the serum vitamin D level was 25.77ng/ml, the FT3/FT4 ratio was the highest. We were unable to find a nonlinear relationship between vitamin D levels and TSHI, TT4RI.

## 4 Discussion

Our study evaluated the connection between thyroid hormone sensitivity and vitamin D levels using The NHANES database. There are many studies on the role of vitamin D in the occurrence of Thyroid diseases, but we still cannot reach a completely unified conclusion. In our view, we are the first team to study the associations between vitamin D and thyroid hormone sensitivity. TFQI and FT3/FT4 were found to be nonlinearly correlated with vitamin D levels, meaning that both the central and peripheral thyroid hormone sensitivity were nonlinearly correlated with vitamin D. We found a U-shaped relationship between TFQI and vitamin D, while an inverse U-shaped relationship between FT3/FT4 and vitamin D. There was no consistent association between vitamin D levels and TSHI, TT4RI.

Serum vitamin D status is closely related to many thyroid diseases. The research of Chao et al. shows that the level of 25(OH)D in the non-HT group was higher than that in the HT group (17). Similarly, Ma et al. discover that HT or GD sufferers had a higher probability of developing vitamin D deficiency contrasted with the control subjects (18). This result was further proved by Sulejmanovic et al. who announced the conclusions that markedly reduced levels of vitamin D in patients with AITD (15). These findings indicate a close relationship between the onset of AITD and vitamin D deficiency. However, we must admit the fact that our study was unable to establish a link between vitamin D levels and HT prevalence. When comparing the vitamin D deficiency group with other groups, we found no statistical difference in TPOAb and TgAb. The conclusions of two former studies were consistent with our research. Cvek et al. confirmed that statistical differences were not found in serum vitamin D levels in HT case groups compared to other groups (18). A study carried out by Botelho declared that patients with low vitamin D levels were not more likely to develop HT (19).

Our study showed statistically significant differences in FT3 and FT4 among groups with different vitamin D levels. But TSH levels between the three groups were not statistically significant. The conclusions of previous relevant studies were controversial. Sandeep et al. discovered a positive link between 25 (OH) D and FT3, FT4 levels, while TSH showed the opposite trend (20). However, a study conducted by Ke et al. proved a conclusion that vitamin D deficiency was not related to TSH and FT4 in patients with HT (21). The reason for the difference in results may be that we did not group 25(OH)D levels in HT or hypothyroid patients, but in the selected population. Another possible reason is that the sample collection time of vitamin D spanned the spring and summer.

TFQI was originally recommended by Laclaustra et al. to assess the extent of thyroid hormone resistance and its relationship with other diseases. Actually, for the reason that our team is the earliest to investigate the connection between thyroid hormone sensitivity and serum vitamin D levels, there is little comparative data available. In our study, we found a U-shaped relationship between TFQI and serum vitamin D levels, while an inverse U-shaped relationship between FT3/FT4 and serum vitamin D levels. When serum vitamin D levels were more than 25.77ng/ml, the TFQI increased with the increase of vitamin D levels. The inflection point value of the curve between vit D and FT3/FT4 was 25.43 ng/ml. Vit D levels were negatively correlated with FT3/FT4when above this value. More recently, Experts have confirmed that a vitamin D concentration of 30-40 ng/mL is sufficient to maintain bone health, and higher levels of vitamin D do not provide additional benefits. Vitamin D levels above 40ng/mL were not found to have any further benefits for bone health. Similarly, in order to achieve good central and peripheral sensitivity at the same time, vitamin D levels must be maintained within the appropriate range, neither too high nor too low.

The pituitary secreted thyrotropin, which stimulated the thyroid to produce thyroxine (T4) and triiodothyronine (T3), of which T4 was the main hormone produced and released by the thyroid, which was converted to T3 by the deiodination of deiodinase in the peripheral. Vitamin D was involved in this complex process to regulate TH levels in the body (22). Thyroid hormone exerted biological effects by binding to thyroid hormone receptors belonging to the nuclear receptor superfamily, which also included vitamins D Receptor (VDR) (23). High concentrations of vitamin D bound specifically to VDR on thyroid cells, inhibiting thyroid cell proliferation and iodine uptake by thyroid follicular cells by reducing the number of thyroid- stimulating hormone (TSH) receptors and inhibiting the adenylate cyclase-CAMP-protein kinase system in a concentration dependent way (24). This seemed to be able to explain part of our findings. Our study found that when the concentration of vitamin D exceeded 25.77ng/ml, the higher the concentration of vitamin D, the higher the value of TFQI, which represented the central sensitivity of the thyroid. The higher the TFQI value, the lower the central sensitivity of the thyroid, indicating that the HPT axis was less sensitive to changes in FT4. Increased vitamin D bound to VDR on thyroid cells, reducing the number of TSH receptors, resulting in TSH being unable to bind to enough TSH receptors to play a role in promoting Thyroxine production timely, thus making TSH insensitive to changes in peripheral FT4.

Alrefaie et al. proved that vitamin D3 was associated with increased levels of type 2 deiodinase (DIO2) and peripheral conversion of TH (specifically from thyroxin-T4 to triiodothyronine-T3) in multiple tissues of diabetic rats, and vitamin D supplementation could reduce TSH values to normal levels in diabetic rats (25). A negative correlation between 25(OH)D levels and TSH had also been observed in the general population (26). This seemed to contradict our findings. We found that when the vitamin D concentration exceeded 25.43ng/ml, the FT3/FT4 ratio decreased with the increase of VID concentration, indicating that the higher the VID concentration, the lower the peripheral sensitivity of thyroid. However, the human body was a whole, in which the central and peripheral sensitivity of thyroid hormone were synergistic. High concentration of vitamin D could inhibit the secretion of TSH, thus reducing the secretion of T4. 80% of T3 in the human body was transformed by T4 through deiodination. Even with increased deiodinase levels, not enough T4 was converted to FT3 due to reduced thyroid secretion of T4, resulting in lower FT3/FT4, which may be one explanation for our results. Of course, we need larger and better designed experiments to test our hypothesis.

However, the relationship between vitamin D and Thyroid hormones may be mutual. At present, little is known about the effect of Thyroid hormones on vitamin D metabolism. Bouillon et al. found that 1,25-dihydroxyvitamin D3 (the active form of vitamin D) decreased in patients with hyperthyroidism, increased in patients with hypothyroidism, and maintained a relatively normal concentration of vitamin D after thyroid function returned to normal (27). This may be related to the effect of Thyroid hormones on calcium absorption in the intestine, but the specific mechanism needs to be confirmed by further studies.

The major highlight of this survey is that we use the NHANES database, which includes data of participants from the whole nation. Due to the large enough sample capacity, we can get more trustworthy conclusion of the U shape relationship between vitamin D and thyroid hormone sensitivity. In addition, confounding factors were taken into account in our analysis. The primary shortcomings of this survey are listed below (1):We cannot speculate on the causal relationship between Thyroid hormone sensitivity and vitamin D levels because this is a retrospective cross-sectional study; (2) On account of the restrictions of NHANES data, some significant variables associated with vitamin D such as seasonal changes, geographical locations and light duration are not available; (3) Some variables such as physical activities, drinking behavior, and smoking behavior are self-judged and therefore the accuracy cannot be fully guaranteed.

## 5 Conclusion

In the US population, our study concluded that FTQI and FT3/FT4 were U-shaped or inverse-U-shaped with serum vitamin D levels respectively after several adjustments. Therefore, FTQI and FT3/FT4 are considered indicators of the complex relationship between thyroid hormone resistance and vitamin D metabolism. In the future, more complex prospective investigations are needed to confirm these findings and find a causal link between them.

## Data availability statement

The original contributions presented in the study are included in the article/**Supplementary Material**, further inquiries can be directed to the corresponding author/s.

## Ethics statement

The studies involving humans were approved by The NCHS Research Ethics Review Board. The studies were conducted in accordance with the local legislation and institutional requirements. The participants provided their written informed consent to participate in this study. Written informed consent was obtained from the individual(s) for the publication of any potentially identifiable images or data included in this article.

## Author contributions

Concept and design: SC, XL, YZ. Data acquisition: WY, ZG. Retrieval and analysis of data: SC, WY. Drafting and interpretation of data: SC. Critical revision: XL, YZ. All authors read and approved the final manuscript.
